# Impairment in the activities of daily living in older adults with and without osteoporosis, osteoarthritis and chronic back pain: a secondary analysis of population-based health survey data

**DOI:** 10.1186/s12891-016-0994-y

**Published:** 2016-03-28

**Authors:** Tanja Alexandra Stamm, Karin Pieber, Richard Crevenna, Thomas Ernst Dorner

**Affiliations:** Section for Outcomes Research, Center for Medical Statistics, Informatics, and Intelligent Systems, Medical University of Vienna and Division of Rheumatology, Department of Internal Medicine III, Medical University of Vienna, Spitalgasse 23, 1090 Vienna, Austria; Department of Physical Medicine and Rehabilitation, Medical University of Vienna, Vienna, Austria; Institute of Social Medicine, Centre for Public Health, Medical University of Vienna, Vienna, Austria

**Keywords:** Activities of daily living, Population-based study, Occupational therapy

## Abstract

**Background:**

Independence in performing activities of daily living (ADLs) is a central aspect of functioning. Older adults frequently experience impairments and limitations in functioning in various life areas. The aim of this survey was to explore the limitations in the ADLs in older adults in a population-based survey in Austria.

**Method:**

A population-based cross-sectional study in 3097 subjects aged ≥65 years who were included in the Austrian health interview survey was performed. Descriptive statistics were used to calculate frequencies of problems in the ADLs. A principal component analysis was applied to analyze the main dimensions of 19 ADL items. Binary logistic regression models were used with the ADL dimensions as the dependent variables and osteoarthritis, chronic back pain, osteoporosis, sex, education level, anxiety or depression, age and pain intensity as independent variables.

**Results:**

People with musculoskeletal conditions were significantly more often affected by ADL problems than people without these diseases. The ADL domain which caused problems in the highest proportion of people was “doing heavy housework” (43.9 %). It was followed by the ADL domains “bending or kneeling down” (39.3 %), “climbing stairs up and down without walking aids” (23.1 %), and “walking 500 m without walking aids” (22.8 %). The principal components analysis revealed four dimensions of ADLs: (1) intense “heavy burden” ADLs, (2) basic instrumental ADLs, (3) basic ADLs and (3) hand-focused ADLs. The proportion of subjects who had problems with the respective dimensions was 58.2, 29.2, 23.0, and 9.2 %. Anxiety/depression (greatest effect), followed by the chronic musculoskeletal disease itself, female sex, higher age and pain intensity were significant predictors of ADL problems.

**Conclusion:**

This population-based survey indicates that older people have considerable ADL problems. More attention should be paid to the high impact of pain intensity, anxiety and depression on ADLs.

## Background

Worldwide, musculoskeletal diseases have major individual, societal, and economic implications. The most common musculoskeletal diseases, osteoarthritis, osteoporosis, and back pain lead to impairments in quality of life and in the activities of daily living (ADLs); as a consequence, they further lead to dependency, institutionalisation and increased health-care costs [1]. These musculoskeletal diseases have been defined as one of the major health priorities both on international level [2, 3] as well as on national level [4, 5].

Independence in the ADLs is a central aspect of functioning. Older adults with and without musculoskeletal complaints frequently experience impairments and limitations in functioning in various life areas [2]. Functioning is a comprehensive concept which includes different types of activities, such as self-care, productivity, leisure and rest, as well as the context in which an activity is performed. The most commonly used conceptual framework to describe functioning that includes all these factors is the World Health Organisation (WHO) International Classification of Functioning Disability and Health (ICF) [6]. In the ICF, functioning is defined as the interplay of the so-called ICF components *body functions, body structures, activities* and *participation,* as well as *environmental* and *personal factors*.

In qualitative studies, people with different types of arthritis described ADLs including self-care as important “problem areas” of all activities performed during a normal day [7–10]. Common symptoms of musculoskeletal conditions, such as pain, morning stiffness, lack of energy and fatigue, but also the lack of support and understanding by other people lead to ADL problems. While these qualitative studies allow us to better understand the life world of other people and to describe potential causes of problems from an inside perspective, population-based surveys are needed to demonstrate the number of people having certain ADL problems. Moreover, if ADL problems increase, they raise the need for additional support and costs for the individual as well as for health and social care institutions [1]. To estimate costs of health and social care, data are needed on (problems with) ADLs of older adults. Furthermore, personal (e.g. sex) and socio-economic factors, as well as comorbidities, pain intensity and anxiety may influence ADL abilities [11]. As health and social care systems differ between countries, Austrian data allow us to describe the current situation in our country, but also to compare our data to other countries. Apart from increased costs, older adults who stop performing ADLs lose the ability to perform these activities independently themselves [12, 13].

The aim of this survey was therefore to explore the limitations in ADLs in older adults with and without osteoporosis, osteoarthritis and chronic back pain (CBP) in a population-based survey in Austria including a discussion on the consequences for society.

## Methods

A population-based cross-sectional study in 3097 subjects aged ≥65 years who were included in the Austrian health interview survey was performed.

### Participants

The database used for our analysis was the Austrian Health Interview Survey (AT-HIS) 2006–07 [14]. This survey was commissioned by the Austrian Federal Ministry of Health, Family and Youth (in German: *Bundesministerium für Gesundheit, Familie und Jugend*) and was carried out by Statistics Austria. The survey is the latest edition of a repetitively performed micro-census of a representative sample of the entire Austrian population, regardless of health status, with the aim to gain knowledge about subjective health, health behaviour, and utilisation of the health care system. The sample was stratified by geographic region, with the same number of subjects being included from each region. The subjects were interviewed between March 2006 and March 2007 by trained interviewers. The gross sample size was 25,130 people, older than 15 years, of which 24,509 were reached. 9,656 subjects were excluded. Out of them 5,709 subjects refused or broke off the interview, 3,308 were excluded due to difficulties in contacting them or because of deficiency in the German language, and in 639 cases there was an insufficiency in data quality. The data of a total of 15,474 subjects was eligible for analysis, thus the response rate was 63 %. The interviews were conducted face-to-face. The questionnaire was designed based on the European Core Health Interview Survey (EC-HIS) [15] and has been adapted for Austria by an expert panel. In order to account for the stratification of the sample the data were weighted by geographic region, age and sex. Missing values were systematised. For this analysis, only data of people older than 65 years were used. Thus, data of 1259 men and 1838 women were eligible for analysis.

### Assessment and survey questions

The musculoskeletal diseases osteoarthritis, CBP, and osteoporosis were assessed with the questions: “Have you ever had osteoarthritis (abrasion or damage of joints), arthritis (inflammation of joints), or rheumatic joint diseases?”, “Have you ever had chronic pain in your spinal column (low back, neck or upper back)?” and “Have you ever had osteoporosis (decrease of bone mass)?”

For the assessment of ADLs, 19 different questions of the AT-HIS were used (Table 1).

**Table 1:** Assessment of ADLs

Interview questions	Scoring
1: Are you able to walk without walking aids for 500 m?	Questions 1 to 7 could be answered by “yes” or “no”.
2. Are you able to climb stairs up and down without walking aids? (Walking aids here included also the use of the handrails.)	
3: Can you use your fingers to grab and use small things like a pencil without problems?	
4: Can you turn on a tap or unscrew the lid of an instant coffee can without any aids without a problem?	
5: Are you able to stretch your hand that you can shake hands without problems?	
6: Are you able to bend or kneel down without a problem?	
7: Are you able to lift and carry a shopping bag with 5 kg without a problem?	
8: Do you usually have any problems to eat independently?	Questions 8 to 19 could be answered by “yes”, “no”, and “unsure”. The answers “yes” and “unsure” were merged together as “having a problem”.
9: Do you usually have problems to sit down on the bed or on a chair and stand up without help?	
10: Do you usually have problems to put on or take off clothes?	
11: Do you usually have problems to go to the toilet?” (Going to the toilet included take off and put on clothes, sit down and get up from the toilet, keep clean or manage a urinary catheter, urostomy or colostomy.)	
12: Do you usually have problems to take a bath or a shower?	
13: Do you usually have problems to prepare your food on your own?	
14: Do you usually have problems to use the telephone independently?	
15: Do you usually have problems to manage your shopping independently?	
16: Do you usually have problems to wash clothes independently?	
17: Do you usually have problems to do light house work independently?	
18: Do you usually have problems to do sometimes heavy house work independently? (Examples for heavy house work were sliding heavy furniture, making spring-cleaning, clean windows, and washing up the floor.)	
19: Do you usually have problems to manage your financial affairs independently?” (Those included withdrawal money or filling in a payment form.)	
Final scoring of ADL questions	All 19 variables were dichotomised with 0 “having no problem” and 1 “having a problem”.

### Analyses

Descriptive statistics were used to calculate frequencies of problems in the ADLs. As co-variables in regression analyses sex, age, education level, pain intensity and presence of anxiety or depression were used. Age was grouped into steps of five years. Level of education was measured as ordinal variable with three levels of education: primary education (up to the age of 15 years), secondary education (apprenticeship or secondary school), and tertiary education (university or any other vocational training). Pain was assessed with the question “Did you suffer from severe pain in one or more than one body site during the last 12 months?”. If this was answered with “yes”, the respondents were shown a picture of the body with 14 different body sites and asked to show the region or regions in which they experienced the pain and for each body site with pain to indicate the intensity of pain on a 10-part visual analogue scale. The highest score in anybody site was used as co-variable in our analysis. Anxiety or depression was assessed with the question “Have you been suffering from anxiety or depression?”.

Bivariate analyses were undertaken by means of cross-tabs, and group differences were assessed with the Pearson’s Chi^2^-test. A procedure factor analysis (principal components analysis) was applied to analyse the main dimensions of the 19 items of ADL [16]. The internal consistency of these dimensions was determined by a reliability analysis (Cronbach’s Alpha). The dichotomous ADL dimensions were then computed by 0 no deficit in the ADLs of the dimensions, and 1 having a deficit in any/at least one of the ADL items of the dimension. Binary logistic regression models were applied with the ADL dimensions as the dependent variables. The musculoskeletal diseases osteoarthritis, CBP, and osteoporosis, sex, the education level, and anxiety or depression were used as categorical, and age (in five year intervals) and pain intensity as metric independent variables in the logistic regression analyses. The results of all logistic regression models are presented as odds ratios with 95 % confidence intervals. Nagelkerkes’ R^2^ is presented as a measure of model-fit. Calculations were done using the procedure of SPSS 20 [IBM].

## Results

In people aged 65 years and older, representative for the Austrian population of this age, 36.1 % of the population reported to have one of the three musculoskeletal diseases (Table 2), 23.1 % were suffering from two of them and 9.2 % were affected by all three of the diseases according to their own statements. Only 31.5 % of the population aged 65 years and older had none of them. Table 2 depicts socio-demographic characteristics of all participants.

**Table 2:** Sociodemographic characteristics of the participants

Sociodemographic characteristics	*N*	%
Sex		
Male	1259	40.7
Female	1838	59.3
Age		
Lower than 75 years	1657	53.5
Higher than 75 years	1439	46.5
Education		
Primary education	1428	46.1
Secondary education	1512	48.8
Tertiary education	157	5.1
Pain		
Severe pain in the last 7 days	1350	43.6
No severe pain in the last 7 days	1747	56.4
Anxiety or depression		
Yes	347	11.2
No	2750	88.8
Health condition(s) – self-reported		
Osteoarthritis	1268	41.0
Chronic back pain	1566	50.6
Osteoporosis	570	18.4
Osteoarthritis AND Chronic back pain	795	25.7
Osteoarthritis AND Osteoporosis	369	11.9
Chronic back pain AND Osteoporosis	403	13.0

The principal components analysis revealed four major dimensions of ADLs (Table 3). One dimension comprised ADL items, in which heavy burdens or far distances have to be managed, so we named it “intense ADLs”. A second dimension consisted of basic *instrumental activities of daily living (IADLs)* like washing clothes, preparing food or doing light house work and we labelled it “basic IADLs”. The third dimension was built with basic activities of daily living like personal hygiene, eating or dressing (“basic ADLs”). A fourth dimension contained items in which the hand and fingers are used (”hand-focused ADLs”). According to the Cronbach’s alpha the first three dimensions showed an excellent, the fourth a fair reliability (Table 3).

**Table 3:** Dimensions of activities of daily living according to a rotated factor analysis

	Dimension 1	Dimension 2	Dimension 3	Dimension 4
Title	Intense ADLs	Basic IADLs	Basic ADLs	Hand-focused ADLs
Components	1 Lift and carry a shopping bag with 5 kg 2 Bend or kneel down 3 Climbing stairs up and down without walking aids 4 Walking 500 m without walking aids 5 Do regularly heavy house work	1 Wash clothes independently 2 Prepare food 3 Do regularly light house work 4 Manage financial affairs independently 5 Make telephone calls independently 6 Shopping	1 Go to the toilet 2 Eat independently 3 Put on and take off clothes 4 Sit down on a chair or bed and stand up 5 Take a bath or a shower	1 Stretch hand and shake hands 2 Turn on a tap or unscrew a can 3 Use fingers to grab and use small things
Description	ADL items, in which heavy burdens or far distances have to be managed	ADL items, which are necessary to live independently in the community	ADL items, which consist of basic self-care tasks	ADL items, in which the hand and fingers are used that focus on shaking, turning and handling small gadgets
Cronbach’s alpha	0.869	0.829	0.847	0.597

58.2 % of the total analysed population had problems in the intense ADLs, 29.2 % in basic IADLs, 23.0 % in basic ADLs and 9.2 % in hand-focussed ADLs. All four dimensions were significantly more often influenced in subjects with osteoarthritis, CBP or osteoporosis (Table 4; Table 5 shows the data for the combinations of the three health conditions).

**Table 4:** Proportion (percent) of 3097 community-dwelling subjects who reported problems or deficits in various activities of daily living (ADLs)

	Osteoarthritis			Chronic back pain			Osteoporosis		
ADL problems/deficits (%)	Yes	No	*p*-value	Yes	No	*p*-value	Yes	No	*p*-value
Walking 500 m without walking aids	32.3	16.1	<0.001	26.1	19.4	<0.001	35.4	19.9	<0.001
Climbing stairs up and down without walking aids	32.9	16.2	<0.001	27.3	18.7	<0.001	37.5	19.8	<0.001
Use fingers to grab and use small things	8.2	3.6	<0.001	6.3	4.6	0.032	10.4	4.4	<0.001
Turn on a tap or unscrew a can	8.1	3.8	<0.001	6.9	4.3	0.002	10.2	4.6	<0.001
Stretch hand and shake hands	3.2	0.9	<0.001	2.5	1.2	0.010	3.5	1.5	0.001
Bend or kneel down	57.3	26.8	<0.001	48.0	30.4	<0.001	58.1	35.0	<0.001
Lift and carry a shopping bag with 5 kg	44.8	22.9	<0.001	37.3	26.3	<0.001	53.2	27.0	<0.001
Eat independently	7.6	6.5	0.219	7.0	6.9	0.912	9.8	6.3	0.003
Sit down on a chair or bed and stand up	14.9	9.6	<0.001	13.8	8.5	<0.001	18.6	9.5	<0.001
Put on and take off clothes	16.6	10.4	<0.001	14.9	10.9	0.001	18.1	11.8	<0.001
Go to the toilet	8.2	6.8	0.154	7.5	7.3	0.758	10.5	6.7	0.002
Take a bath or a shower	22.2	12.8	<0.001	19.2	14.1	<0.001	25.8	14.6	<0.001
Prepare food	21.7	15.3	<0.001	19.7	16.1	0.010	23.3	16.9	0.003
Make telephone calls independently	6.4	5.1	0.140	5.7	5.6	0.937	7.4	5.3	0.049
Go shopping independently	27.9	15.1	<0.001	23.5	17.2	<0.001	31.1	17.9	<0.001
Wash clothes independently	24.8	17.4	<0.001	22.0	18.9	0.037	24.4	19.6	0.010
Do regularly light house work	21.4	13.9	<0.001	19.6	14.2	<0.001	24.4	15.2	<0.001
Do regularly heavy house work	57.4	34.6	<0.001	49.2	38.6	<0.001	62.1	39.8	<0.001
Manage financial affairs independently	15.4	8.9	<0.001	11.9	11.1	0.496	16.4	10.4	<0.001
ADL dimension 1*	74.5	46.9	<0.001	65.9	50.3	<0.001	77.5	53.8	<0.001
ADL dimension 2*	35.6	24.7	<0.001	32.2	26.1	<0.001	37.9	27.2	<0.001
ADL dimension 3*	29.7	18.3	<0.001	26.2	19.7	<0.001	34.6	20.3	<0.001
ADL dimension 4*	13.6	6.1	<0.001	11.2	7.1	<0.001	15.8	7.7	<0.001

The p-values refer to differences between those with and without the respective musculoskeletal disorder and were calculated with the Pearson’s Chi^2^ test

*ADL dimensions are described in Table 3

**Table 5:** Combinations of the three health conditions

	Osteoarthritis and chronic back pain			Osteoarthritis and osteoporosis			Chronic back pain and osteoporosis		
ADL problems/deficits (%)	Yes	No	*p*-value	Yes	No	*p*-value	Yes	No	*p*-value
Walking 500 m without walking aids	34.3	18.7	<0.001	43.6	19.9	<0.001	38.4	20.4	<0.001
Climbing stairs up and down without walking aids	35.4	18.8	<0.001	44.4	20.2	<0.001	40.0	20.5	<0.001
Use fingers to grab and use small things	9.6	4.0	<0.001	12.5	4.5	<0.001	9.9	4.8	<0.001
Turn on a tap or unscrew a can	9.9	4.1	<0.001	12.5	4.7	<0.001	10.7	4.8	<0.001
Stretch hand and shake hands	4.4	1.0	<0.001	4.1	1.6	0.001	3.5	1.6	0.011
Bend or kneel down	61.3	31.7	<0.001	68.8	35.3	<0.001	61.6	35.9	<0.001
Lift and carry a shopping bag with 5 kg	46.7	26.7	<0.001	59.1	28.2	<0.001	55.3	28.3	<0.001
Eat independently	7.9	6.6	0.223	10.6	6.5	0.004	9.9	6.5	0.013
Sit down on a chair or bed and stand up	17.1	9.2	<0.001	20.7	9.9	<0.001	19.1	10.0	<0.001
Put on and take off clothes	18.6	11.0	<0.001	19.5	12.1	<0.001	18.9	12.1	<0.001
Go to the toilet	7.9	7.2	0.509	10.6	7.0	0.012	9.2	7.2	0.150
Take a bath or a shower	24.2	14.0	<0.001	29.9	14.9	<0.001	25.8	15.3	<0.001
Prepare food	23.6	15.9	<0.001	26.4	16.8	<0.001	23.8	17.0	0.001
Make telephone calls independently	6.9	5.2	0.073	6.8	5.5	0.313	8.7	5.2	0.005
Go shopping independently	30.7	16.8	<0.001	35.2	18.4	<0.001	33.7	18.3	<0.001
Wash clothes independently	27.0	18.2	<0.001	28.2	19.4	<0.001	25.7	19.7	0.005
Do regularly light house work	24.7	14.3	<0.001	27.7	15.5	<0.001	25.8	15.6	<0.001
Do regularly heavy house work	59.0	38.7	<0.001	68.3	40.6	<0.001	62.3	41.2	<0.001
Manage financial affairs independently	16.1	9.9	<0.001	17.3	10.7	<0.001	17.6	10.6	<0.001
ADL dimension 1*	69.2	46.3	<0.001	79.1	48.5	<0.001	73.7	48.9	<0.001
ADL dimension 2*	39.7	25.6	<0.001	43.6	27.2	<0.001	40.9	27.5	<0.001
ADL dimension 3*	64.2	36.2	<0.001	71.2	39.7	<0.001	66.3	40.0	<0.001
ADL dimension 4*	16.6	6.6	<0.001	19.0	7.8	<0.001	16.6	8.1	<0.001

This table is similar to Table 2, but combines two health conditions, respectively, showing proportions (percent) of 3097 community-dwelling subjects who reported problems or deficits in the activities of daily living (ADLs). The p-values refer to differences between those with and without the respective musculoskeletal disorders and were calculated with the Pearson’s Chi^2^ test

*ADL dimensions are described in Table 3

In all participants taken together, the ADL which caused the problem in the highest proportion of subjects was “doing heavy housework” (43.9 %), followed by “bend or kneel down” (39.3 %), “climbing stairs up and down without walking aids” (23.1 %), and “walking 500 m without walking aids” (22.8 %). In Table 4, the proportion of subjects with and without musculoskeletal diseases who reported deficits in various ADLs is shown. All assessed items of ADL were significantly more often impaired in people with osteoporosis compared to those without osteoporosis. Furthermore in people with osteoarthritis and in people with CBP, a large number of ADLs were significantly more often affected compared to those without these diseases (Table 4). Compared to people with osteoarthritis, a higher proportion of people with osteoporosis reported deficits in all ADL items. Furthermore, a higher proportion of people with osteoarthritis reported ADL problems than people with CBP. Table 5 shows ADL problems/deficits in combinations of the three health conditions.

In Table 6, the factors that were found to influence the four dimensions of ADLs in the multivariate logistic regression analysis are presented. Osteoarthritis was associated with a 68 % higher chance of a detraction of intense ADLs, and with a 32 % higher chance of impairment in hand-focussed ADLs. After adjustment, CBP was not associated with problems in any dimension of ADL. Osteoporosis increased the chance of detraction in intense ADLs by 45 % and of basic IADLs by 37 %.

**Table 6:** Logistic regression results (odds ratios, ORs) of having deficits in activities of daily living of various dimensions

		Dependent variables							
		Dimension 1		Dimension 2		Dimension 3		Dimension 4	
		Intense ADLs		Basic IADLs		Basic ADLs		Hand-focussed ADLs	
		OR	95 % CI	OR	95 % CI	OR	95 % CI	OR	95 % CI
Independent variables	Osteoarthritis	1.68	1.39–2.03	1.09	0.90–1.32	0.98	0.80–1.20	1.32	1.00–1.75
	Chronic back pain	1.19	0.99–1.42	0.94	0.78–1.14	0.95	0.78–1.16	1.03	0.78–1.36
	Osteoporosis	1.45	1.11–1.88	1.37	1.08–1.73	1.24	0.97–1.57	1.25	0.92–1.71
	Female sex (ref: male)	1.24	1.03–1.50	0.41	0.33–0.50	1.07	0.86–1.32	1.02	0.76–1.38
	Age (5 year intervals)	1.86	1.74–2.00	1.80	1.68–1.93	1.79	1.66–1.92	1.47	1.34–1.62
	Primary education (ref: tertiary)	1.17	0.79–1.74	0.90	0.60–1.36	0.96	0.60–1.55	1.24	0.59–2.62
	Secondary education (ref: tertiary)	0.85	0.58–1.25	0.59	0.39–0.88	0.73	0.46–1.18	1.21	0.58–2.55
	Pain intensity (per point)	1.26	1.22–1.31	1.15	1.12–1.18	1.18	1.14–1.22	1.15	1.11–1.20
	Anxiety or depression	2.63	1.90–3.64	2.31	1.79–2.99	2.41	1.86–3.14	2.84	2.09–3.86
	Nagelkerke’s R^2^	0.378		0.250		0.257		0.166	

Female sex was associated with an increased risk of deficits in intense ADLs, but with a decreased risk of deficits in basic IADLs. Age was associated with an increased risk of detraction in all four dimensions of ADL. The level of education had mostly no influence on ADL performance; however, secondary education was, compared to tertiary education associated with a lower risk for impairment in basic IADLs like household keeping. Pain intensity increased the chance of deficits of all dimension of ADL. The greatest effect on all dimensions of ADLs was found regarding self-reported anxiety of depression. Those mental diseases doubled to tripled the risk for impairment in the respective dimensions of ADL.

## Discussion

This study showed that older adults with and without osteoarthritis, CBP and osteoporosis in a population-based survey in Austria experienced considerable problems in performing their ADLs - despite that they all were “receiving” the “normal” Austrian health care. Only 40 % of the people aged ≥65 years had no ADL limitations in intense ADLs (dimension 1). This may show that there is additional need for interventions that focus on ADLs. The number of older adults with musculoskeletal complaints who experience problems in household activities and mobility in this study clearly demonstrates the need for functional assessment and corresponding interventions to prevent worsening and further ADL-, health and social problems which would consequently raise also costs for the individual and society. This group of people should therefore be a priority target for specialists of physical medicine and rehabilitation and occupational therapists, as well as other health professionals who aim to achieve optimal functioning in daily life. Physicians, nurses and therapists may be recommended to screen older adults for ADL problems and in case refer them to these specialists. If ADL problems are not taken seriously e.g. due to a musculoskeletal disease, these problems consequently increase leading to additional costs for health and social care [17].

Interestingly, similar activities were experienced more often as problematic across these three health conditions. Furthermore, the ADLs which caused problems in the highest proportion of subjects are in two main “areas”: mobility and housework. The following two chapters within the ICF component *activities and participation* include all these activities: “Chapter 4 Mobility” and “Chapter 6 Domestic life”. *Doing heavy housework* can be linked to the ICF category “d640 Doing housework”, *Bend or kneel down* to “d410 Changing basic body position”, *Climbing stairs up and down without walking aids* to “d4551 Climbing” and *Walking 500 m without walking aids* to “d4500 Walking short distances (less than a kilometre)”. Dimensions 1 (Intense ADLs) and 4 (Hand-focused ADLs) in Table 3 deal also with mobility and housework because the ICF “Chapter 4 Mobility” includes also hand-based activities such as fine hand use (d440) or lifting and carrying objects (d430). Hand-based activities were one separate dimension in the factor analysis on ADL problems in these 3 musculoskeletal diseases. While this is obvious in osteoarthritis (osteoarthritis is most common in hips, knees and hands), back pain and osteoporosis do not mainly affect the hands. Those two areas (and corresponding ICF chapters) mobility and domestic life could therefore be important areas to consider in the assessment of older adults with different musculoskeletal complaints.

In Table 4, ADL deficits were significantly more often reported by people with the three musculoskeletal conditions (compared to people without the respective conditions), while in Table 6, this relationship between ADL problems and diseases appears to be considerably reduced. We therefore concluded that the ADL deficits are partly related to the factors for which we controlled in the multivariate models, such as pain, mental health [18–20] etc. However, some relationships such as between osteoarthritis and ADL deficits in dimension 1 and 4 or osteoporosis and ADL deficits in dimension 1 and 2 appear also in the models - indicating that ADL problems in these dimensions are related to these conditions and not influenced by the other factors controlled for in the models.

Female sex was associated with increased deficits in intense ADLs (dimension 1) and decreased deficits in basic ADLs (dimension 2). This may show traditional gender differences in the performance of ADLs in Austria, in that women still take over more household tasks and are more likely limited in terms of “heavy” activities such as being included in dimension 1. In one of our recent qualitative studies, the life stories of women and men with arthritis reflected how contextual factors (such the healthcare system, the support of families and social and cultural values) shaped their everyday activities. Men for example had more difficulties to develop a non-paid-work related role in their lives than women [21]. Especially dimension 2 (basic IADLs) in our study includes several activities that are primarily done by women in Austria, such as washing clothes, preparing food and regular light housework. This dimension seems to be an important aspect for the possibility of living independently in the community, whereas the activities in dimension 1 could be taken over by social networks.

While the ICF is a comprehensive framework including a classification of functioning in daily life, other definitions that characterise an activity as either ADL or IADL exist. Basic ADLs (BADLs) consist of self-care tasks, including bathing and showering (washing the body), bowel and bladder management (recognizing the need to relieve oneself), dressing, eating (including chewing and swallowing), feeding (setting up food and bringing it to the mouth), functional mobility (moving from one place to another while performing activities), personal device care, personal hygiene and grooming (including washing hair), sexual activity and toilet hygiene (completing the act of relieving oneself) [22]. Instrumental activities of daily living (IADLs) are not necessary for fundamental functioning, but they let an individual live independently in a community. Examples are housework, taking medications as prescribed, managing money, shopping for groceries or clothing, use of telephone or other form of communication, using technology and transportation within the community [23].

Participants with only secondary education were less likely to have ADL problems in dimension 2, while age and pain were clearly associated with more ADL problems in all dimensions. While age without any health problems also increases ADL problems, the highest ORs in our study were associated with anxiety and depression. However, we cannot determine the causal relationship because anxiety and depression can lead to increased ADL problems, but also vice-versa, ADL problems leading to increased anxiety and depression.

Chapter 5 of the ICF “Self-care” was mostly represented in dimension 3 and presented a dependency on age, anxiety and depression. The age-dependent loss of independent eating, dressing or toileting is an important aspect because of the rising age of the population.

A strength of our study is the fact that the survey was population-based. Thus, selection bias that would occur, if only participants living in clinical or nursing home settings were interviewed, could be avoided. Some limitations of our study have to be mentioned. Data were gathered in the Austrian Health Survey and we could not influence the list of activities that were included. Also there was no question on the priority of the activities, nor a question asking each individual to name one primary ADL-problem. Diagnoses were self-reported; people thus may have overestimated certain symptoms, such as joint pain, and on the other hand may not have known about other health conditions, such as osteoporosis. As Nagelkerke’s R2 ranged between 0.166 and 0.378, other factors may have contributed to ADL problems that were not captured in this population-based survey, such as gross and fine motor skills, balance, strength and sensory functions, as well as personal and environmental factors. All questions focused mainly on activities, but not on body functions or structures, nor on environmental factors influencing the performance of these activities. Furthermore, the question for anxiety and depression is very personal and will therefore be not always honestly answered in such an interview survey.

Cronbach’s alpha in the 4^th^ dimension was low. A possible reason can be that all three items in the 4^th^ dimension included two concepts to score in one item (stretch hand AND shake hands; turn on a tap OR unscrew a can; use fingers to grab AND use small things) – this is difficult to sore and participants may have understood (and consequently scored) these items differently [24].

## Conclusion

This population-based survey indicates that people with self-reported osteoporosis, osteoarthritis and CBP have considerable ADLs problems despite “normal” Austrian health care. Older adults with musculoskeletal complaints could be a special target group for ADL specialists such as physical medicine and rehabilitation physicians and occupational therapists. More attention should be paid to the high impact of pain (intensity) as well as anxiety and depression on ADLs.

### Ethics approval

The secondary analysis of the AT-HIS database which was used for this study was approved by the Ethics Committee of the Medical University Vienna (EC# 770/2011).

### Data availability

All relevant raw data will be freely available to any scientist wishing to use them for non-commercial purposes, without breaching participant confidentiality.

## Abbreviations

ADLs: Activities of daily living
CBP: Chronic back pain
WHO: World Health Organisation
ICF: International Classification of Functioning Disability and Health
AT-HIS: Austrian Health Interview Survey
